# Fertility-Sparing Treatment for Early-Stage Cervical Cancer ≥ 2 cm: Can One Still Effectively Become a Mother? A Systematic Review of Fertility Outcomes

**DOI:** 10.1245/s10434-023-13542-z

**Published:** 2023-06-01

**Authors:** Carlo Ronsini, M. C. Solazzo, R. Molitierno, P. De Franciscis, F. Pasanisi, L. Cobellis, N. Colacurci

**Affiliations:** Department of Woman, Child and General and Specialized Surgery, University of Campania “Luigi Vanvitelli”, Naples, Italy

**Keywords:** Cervical cancer, Fertility sparing treatment, Trachelectomy, Birth rat, Pre-term rate, Pregnancy rate

## Abstract

**Background:**

Fertility-sparing treatments (FSTs) have played a crucial role in the management of early-stage cervical cancer (ECC); however, there is currently no standard of care for women with ECC ≥ 2 cm who wish to preserve their fertility. The current orientation of the scientific community comprises upfront surgical techniques and neoadjuvant chemotherapy (NACT) followed by minor surgery such us conization. However these approaches are not standardized. This systematic review aimed to collect the evidence in the literature regarding the obstetric outcomes of the different techniques for applying FSTs in ECC ≥ 2 cm.

**Methods:**

A systematic review was performed in September 2022 using the Pubmed and Scopus databases, from the date of the first publication. We included all studies containing data regarding pregnancy, birth, and preterm rates.

**Results:**

Fifteen studies fulfilled the inclusion criteria, and 352 patients were analyzed regarding fertility outcomes. Surgery-based FST showed the pregnancy rate (22%), birth rate (11%), and preterm rate (10%). Papers regarding FST using the NACT approach showed a pregnancy rate of 44%, with a birth rate of 45% in patients who managed to get pregnant. The preterm rate amounted to 44%, and pregnancy rates and birth rates were significantly different between the two groups (*p* < 0.001).

**Conclusion:**

Fertility preservation in patients with ECC > 2 cm is challenging. The endpoint for evaluating the best treatment should include oncological and fertility outcomes together. From this prospective, NACT followed by less radical surgery could be a reasonable compromise.

**Supplementary Information:**

The online version contains supplementary material available at 10.1245/s10434-023-13542-z.

Cervical cancer is the fourth most common malignancy in women worldwide. Almost 40% of women with cervical cancer are diagnosed between the ages of 20 and 44 years, with the disease confined to the cervix in approximately 46% of cases.^1^ On the other hand, the average age of a woman at first pregnancy is increasing, making it common for patients to be diagnosed with early-stage cervical carcinoma (ECC) who have not yet completed their reproductive expectations. Therefore, fertility-sparing treatments (FSTs) have been considered an alternative to the ‘standard’ radical hysterectomy to preserve women’s fertility and quality of life.^2^ ECC management is controversial, depending on the tumor stage and other risk factors such as tumor size, histotype, grade, and lymphovascular invasion. Radical trachelectomy (RT) combined with pelvic lymphadenectomy (PLND) is the treatment of choice for women with stage 1B1 cervical cancer < 2 cm who wish to preserve their fertility. RT may be performed vaginally, abdominally, or laparoscopically/robotically.^3^ Reviews have confirmed that vaginal RT (VRT) is an oncologically safe option for this type of patient^4^ and have shown that 80% of women can conceive after VRT.^2^ On the other hand, tumor size ≥ 2 cm is an area in which there is less concordance in the literature and less standardization of techniques. Essentially, two approaches are offered to these women: surgical FST and neoadjuvant chemotherapy (NACT) followed by conization FST. In a previous review, we have remarked on the significant heterogeneity present in the clinical management of FST of ECC ≥ 2 cm, focusing mainly on oncological outcomes.^5^ However, once oncological safety is demonstrated, it should be crucial to deeply understand the impact those two approaches have on fertility outcomes. The purpose of this review was to compare the rates of pregnancy, live births, and preterm rates for women with ECC ≥ 2 cm treated with surgical FST or NACT approaches.

## Material and Methods

The methods for this study were specified a priori based on the recommendations reported in the Preferred Reporting Items for Systematic Reviews and Meta-Analyses (PRISMA) statement.^6^ The study was registered in the PROSPERO database for meta-analysis, with protocol number CRD42022329253.

### Search Method

We performed a systematic search for articles regarding fertility outcomes in FST of ECC ≥ 2 cm in the Pubmed and Scopus Databases in September 2022, from the date of the first publication We made no restrictions on country, and considered only studies published in the English-language. Search terms used were ‘fertility sparing’ and ‘cervical neoplasm’ for each database.

### Study Selection

**Fig. 1:**
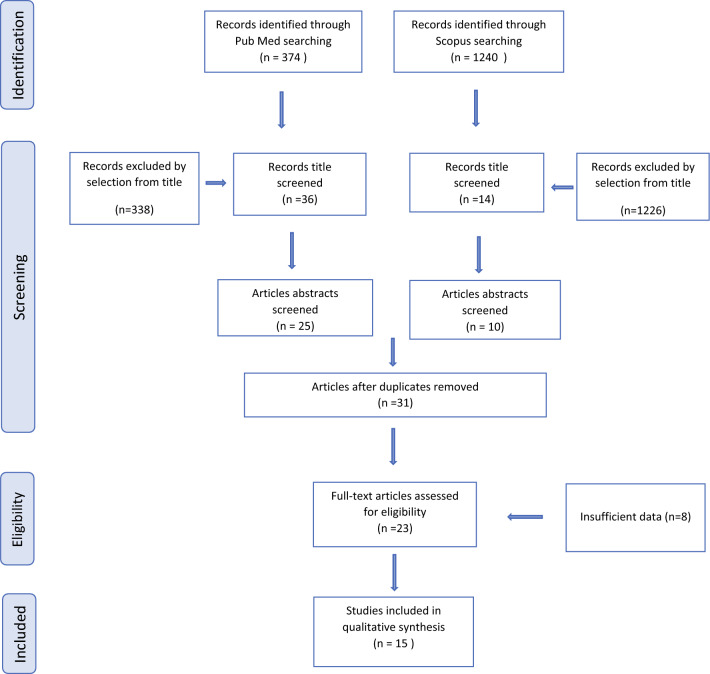
PRISMA flow diagram. *PRISMA* Preferred Reporting Items for Systematic Reviews and Meta-Analyses

Study selection was made independently by MCS and CR, and in case of discrepancies, CR decided on the inclusion or exclusion of a study. Inclusion criteria were (1) studies that included patients with ECC ≥ 2 cm; (2) studies that reported at least one outcome of interest (pregnancy rate, birth rate, preterm rate); and (3) original peer-reviewed articles. We excluded non-original studies, preclinical trials, animal trials, abstract-only publications, and articles in languages other than English. If possible, the authors of studies that were only published as congress abstracts were contacted via email and asked to provide the relevant data. The selected studies and the reasons for exclusion are reported in Fig. 1. All included studies were assessed regarding any potential conflicts of interest.

### Data Extraction and Analysis

MCS and MR extracted data for all relevant series and case reports. Data on the surgical approach to tumors (surgical-FST or NACT-FST) and fertility outcomes, such as the number of patients who attempted to conceive after treatment, pregnancy rate, birth rate, and preterm rate, were extracted. The pregnancy rate was defined as the ratio of patients with at least one pregnancy and the total number of patients who attempted to conceive. The birth rate was defined as the ratio of live-birth deliveries to the total number of patients who attempted to become pregnant; a premature delivery was defined as a delivery < 37 weeks’ gestation (WG). The preterm rate was defined as the ratio of premature deliveries to the total number of pregnancies resulting in live births; however, this activity was hindered by different criteria across papers and a diffused lack of information. Four studies did not specifically report the number of attempted conceptions, and in these cases, the authors considered the total number of patients who underwent successful FST. Chi-square tests were used to compare continuous variables.

### Quality Assessment

We assessed the quality of the included studies using the Newcastle–Ottawa Scale (NOS).^7^ This assessment scale uses three broad factors (selection, comparability, and exposure), with the scores ranging from 0 (lowest quality) to 8 (best quality). Two authors (MR and MLV) independently rated the quality of the studies. Any disagreements were subsequently resolved by discussion or consultation with a third author (CR). The NOS scale is reported in the electronic supplementary material.

## Results

### Study Characteristics

From database screening, 1614 studies were selected. After removing records with no full text, duplicates, and wrong study designs, 23 studies were suitable for eligibility, of which 15 matched the inclusion criteria and were included in the systematic review. Overall, the publication years of the studies ranged from 2013 to 2021. The basic characteristics of the included studies (first author, year of publication, country, study design, study range [years], and the number of participants) are described in Table 1.

**Table 1:** Study characteristics

Study, year	Country	Study design	Study year	FIGO stage	No. of participants
Cao et al.^8^	China	Prospective, case-control, multicentric	2003–2012	IB1	48
De Vincenzo et al.^9^	Italy	Retrospective, observational, monocentric	2014–2018	IB2	9
Deng et al.^10^	China	Retrospective, observational, monocentric	–	IB1 > 2 cm^	45
Guo et al.^11^	China	Retrospective, observational, monocentric	2003–2016	IB1 > 2 cm^	75
Lanowska et al.^12^	Germany	Retrospective, observational, monocentric	2006–2013	IB1 > 2 cm^ IB2	20
Li et al.^13^	China	Retrospective, observational, monocentric	2004–2013	IB1 > 2 cm^	55
Lintner et al.^14^	Hungary UK USA	Retrospective, observational, multicentric	1999–2006	IB1 > 2 cm^ IB2	31
Lu et al.^15^	China	Retrospective, observational, monocentric	2005–2012	IB1 > 2 cm^	7
Marchiole et al.^16^	France	Retrospective, observational, monocentric	2007–2017	IB1 > 2 cm^ IB2 IIA1 > 2 cm^	19
Rendón et al.^17^	Colombia	Retrospective, observational, monocentric	2009–2019	IB1 >2 cm^ IB2^ IIA1 > 2 cm^	23
Robova et al.^18^	Czech Republic	Retrospective, observational, monocentric	2005–2013	IB1 > 2 cm^ IB2^	20
Salihi et al.^19^	Belgium	Retrospective, observational, monocentric	2004–2013	IB1 > 2 cm^ IB2^	5
Tesfai et al.^20^	Netherlands	Retrospective, observational, monocentric	2006–2018	IB–IIA^	15
Wethington et al.^21^	USA	Retrospective, observational, monocentric	2001–2011	IB1	9
Zusterzeel et al.^22^	Netherlands	Retrospective, observational, monocentric	2009–2018	IB2	14

*FIGO* International Federation of Gynecology and Obstetrics

### Outcomes

A total of 395 patients were included in this review. Six of the 15 selected studies presented data regarding fertility outcomes in surgical FST, while the remaining nine studies presented data on FST with the use of NACT. No studies reported data from a direct comparison between these two FSTs. The overall pregnancy, birth, and preterm rates for the surgical FST procedures were 22.2, 11.1, and 10%, respectively. Furthermore, in the NACT group, the pregnancy rate amounted to 44%, and data showed birth and preterm rates of 45.5 and 43.9%, respectively. Pregnancy and birth rates were significantly higher in the NACT group (pregnancy rate 22.2% vs. 44.4%, *p* = 0.0016; birth rate 11.1% vs. 45.5%, *p* < 0.001). On the contrary, preterm births were more frequent in NACT patients (10% vs. 43.9%, *p* = 0.047). The fertility outcomes of two groups are summarized in Table 2.

**Table 2:** Surgical FST and NACT fertility outcomes

Outcomes	Surgical FST	NACT	*p*.
Pregnancy rate	22.2 (20)	44.4 (40)	0.0016
Birth rate	11.1 (10)	45.5 (41)	0.00001
Preterm rate	10 (1)	43.9 (18)	0.047

*FST* fertility-sparing treatment, *NACT* neoadjuvant chemotherapy

### Surgical Fertility-Sparing Treatment (FST) Outcomes

Cao et al.^8^ performed a retrospective comparison between vaginal and abdominal trachelectomy in ECC. A total of 48 patients with ECC > 2 cm were recruited—24 in the VRT group and 24 in the abdominal RT (ART) group. In a mean follow-up period of 20 months, independently from the technique used, only 24 patients attempted to conceive, three of whom had a pregnancy (pregnancy rate 12.5 %). The live birth rate was 12.5%; data regarding the preterm rate are not available.

Deng et al.^10^ enrolled 45 patients with stage IB1 cervical cancer who had tumors larger than 2 cm treated with ART guided by the sentinel lymph node biopsy (SNLB) procedure. After a follow-up period of 45 months, 19 patients tried to conceive (42.2%) and five succeeded, for a total of five pregnancies after surgery (pregnancy rate 26.32%). Of these five pregnancies, one was a term delivery (birth rate 5%, preterm rate 0%), one was a mid-trimester miscarriage, and three were first-trimester miscarriages.

Guo et al.^11^ investigated the oncological safety of ART compared with radical hysterectomy. Seventy-five patients with ECC > 2 cm were recruited and agreed to ART. The follow-up time was 70 months. During this period, 29 women tried to conceive (38.6 %), resulting in five pregnancies (pregnancy rate 17.2%). Among these pregnancies, there were two live births (birth rate 6.9%); the preterm rate was not estimated.

Li et al.^13^ conducted a retrospective review of the oncological, surgical, and obstetric outcomes of patients undergoing ART for ECC ≥ 2 cm. A total of 55 patients preserved their fertility potential. In a mean follow-up period of 30.2 months, nine patients tried to conceive (16.3%); three were successful (33%) but there was only one live birth (birth rate 11%).

Lintner et al.^14^ reported 30 patients with ECC > 2 cm treated with ART plus PLND. These authors reported a median follow-up time of 90 months, during which eight women tried to conceive (23.3%). Three pregnancies led to the delivery of a healthy neonate (pregnancy rate 42.8% and birth rate 42.8%)—one at 28 weeks’ gestation (preterm rate 33.3%) and two at term.

Wethington et al.^20^ reported a case series of nine patients treated with both abdominal and laparoscopic trachelectomy (LRT) and robotic trachelectomy (RRT). In a median follow-up period of 40 months, two women tried to conceive (22.2%), one of whom had a pregnancy (pregnancy rate 50%), but none of them delivered (birth rate and pregnancy rate 0%). Overall, surgical FST techniques showed a pregnancy rate of between 12.5 and 50%, a birth rate between 0 and 42.8%, and a preterm rate of between 0 and 33.3%. The follow-up period ranged from 20 to 90 months on average.

The overall results, derived from recalculation of all the mentioned studies, reported a pregnancy rate of 22.2%, birth rate of 11.1%, and preterm birth of 10%. These results are summarized in Table 3.

**Table 3:** Surgical FST outcomes

	Attempted to conceive/all patients [*n*/*N* (%)]	Pregnancy rate [% (*n*)]	Birth rate [% (*n*)]	Preterm rate [% (*n*)]	Mean FUP (months)
Cao et al.^8^	24/48 (50)	12.5 (3)	12.5 (3)	NR	20
Deng et al.^10^	19/45 (42.2)	26.32 (5)	5 (1)	0	45
Guo et al.^11^	29/75 (38.6)	17.2 (5)^a^	6.9 (2)	NR	70
Li et al.^13^	9/55 (16.3)	33 (3)	11 (1)	0	30.2
Lintner et al.^14^	7/30 (23.3)	42.8 (3)^b^	42.8 (3)	33.3 (1)	90
Wethington et al.^21^	2/9 (22.2)	50 (1)	0	0	44
Total	90/262 (34.3)	22.2 (20)	11.1 (10)	10 (1)	

*FST* fertility-sparing treatment, *FUP* follow-up, *NR* not reported

^a^Five women had eight pregnancies

^b^Three women had four pregnancies

### Neoadjuvant Chemotherapy FST Outcomes

In their retrospective observational study, De Vincenzo et al.^9^ published data on nine patients treated with three cycles of cisplatin and paclitaxel q21 and then treated with cold-knife conization. Among the nine patients, only three patients tried to conceive and two became pregnant, both spontaneously (pregnancy rate and birth rate 66.6%). One patient underwent a cesarean section at 34 weeks 3 days because of preterm premature rupture of membranes (PROMs). The other woman was subjected to a cesarean section at 37 weeks and 2 days because of PROMs and maternal request (preterm rate 50%). Both babies were in good condition. The third patient reported several unsuccessful attempts to become pregnant, likely due a reported cervical stenosis.

Lanowska et al.^12^ reported on the experience of 20 patients treated with NACT followed by VRT. Seven of 20 patients tried to become pregnant and seven pregnancies occurred in five women, with a pregnancy rate of 71.4% and a birth rate of 57.4%. One ectopic pregnancy and one miscarriage occurred. All four babies were born by cesarean delivery and two premature deliveries occurred due to premature rupture of the membranes and vaginal bleeding, respectively (preterm rate 50%).

Marchiole et al.^16^presented a series of seven patients treated with three or four cycles of cisplatin + paclitaxel + ifosfamide with a VRT of completion. The pregnancy rate was 50%. Three women had eight pregnancies; four first trimester miscarriages and one therapeutic abortion at 18 weeks occurred, with a birth rate of 17.6%. All three babies were born prematurely by cesarean delivery (preterm rate 100%).

Lu et al.^15^ successfully treated six women who underwent NACT followed by total LRT. In a median follow-up of 66 months, four women attempted to conceive and two succeeded (pregnancy rate 50%). One patient had a miscarriage in the first trimester and the other patient underwent a cesarean section due to PROMs. The authors reported a birth rate of 25% and a preterm rate of 100%.

Rendón et al.^17^ reported on 23 patients treated with different chemotherapy regimens combined with conization. After a median follow-up period of 47 months, seven women delivered 11 babies and three women delivered twice (pregnancy rate 43.5%). There were four term deliveries, seven preterm births (preterm rate 63.3%), and an ongoing pregnancy at 18 weeks.

In 2014, Robova et al.^18^ reported on data regarding fertility outcomes from 20 patients treated with different types of NACT followed by vaginal simple trachelectomy plus laparoscopic lymphadenectomy. Fertility-sparing procedure was performed in all patients, with a pregnancy rate of 50%; eight women delivered 10 babies, and four premature deliveries (preterm rate 40%).

A subanalysis of the paper by Salihi et al.^19^ showed data from five patients with ECC ≥ 2 cm. In this group, only one pregnancy occurred, with a birth rate of 20%.

Tesfai et al.^20^ presented a series of 19 women treated with ART after neoadjuvant chemotherapy. Three of 15 patients with a successful ART became pregnant and had eight spontaneous pregnancies (pregnancy rate 20%) during the median follow-up period of 73 months. All women delivered at full term via cesarean section (birth rate 40%). One patient terminated two pregnancies due to non-medical reasons.

Finally, Zusterzeel et al.^22^ evaluated fertility outcomes in a series of 14 women treated with NACT followed by VRT and PLND. In a median follow-up period of 50 months, seven women tried to conceive (50%), resulting in four patients having six pregnancies, including two first-trimester miscarriages and three live births born at term. The birth rate was 42.8% and the preterm rate was 0%.

The overall results, derived from recalculation of all the mentioned studies, reported a pregnancy rate of 44%, birth rate of 45.5%, and preterm birth rate of 43.9%. In a median follow-up period of between 23 and 73 months, the application of NACT schemes in 90 patients resulted in 40 pregnancies, 41 live births, and 18 preterm deliveries. These results are summarized in Table 4. Substratification by surgical approach after NACT showed a pregnancy rate of 41.9%, a birth rate of 45.1%, and a preterm rate of 61.5% for conization; 55.0, 50.0, and 36.0% for VRT, respectively; 50.0, 50.0, and 25.0% for minimally invasive RT, respectively; and 20.0, 40.0, and 0% for ART, respectively. These results are summarized in Table 5.

**Table 4:** NACT fertility outcomes

	Attempted to conceive/all patients [*n*/*N* (%)]	Pregnancy rate [% (*n*)]	Birth rate [% (*n*)]	Preterm rate [% (*n*)]	Mean FUP (months)
De Vincenzo et al.^9^	3/9 (33.3)	66.6 (2)	66.6 (2)	50 (1)	37
Lanowska et al.^12^	7/20 (35)	71.4 (5)^a^	57.1 (4)	50(2)	23
Marchiole et al.^16^	6/17 (28.3)	50 (3)^b^	17.6 (3)	100(3)	NR
Lu et al.^15^	4/7 (54.1)	50 (2)	25 (1)	100 (1)	66
Rendón et al.^17^	NR/23	43.5 (10)^c^	47.8 (11)	63.6 (7)	47
Robova et al., 2014^18^	NR/20	50 (10)	50 (10)^d^	40 (4)	42
Salihi et al.^19^	NR/5	20 (1)	20 (1)	0	58
Tesfai et al.^20^	NR/15	20 (3)^e^	40 (6)	0	73
Zusterzeel et al.^22^	7/14 (50)	57.1 (4)^f^	42.8 (3)	0	50
Total	90/130 (69.2)^g^	44.4 (40)	45.5 (41)	43.9 (18)	

*FST* fertility-sparing treatment, *NACT* neoadjuvant chemotherapy, *FUP* follow-up, *NR* not reported

^a^Five women had seven pregnancies

^b^Three women had eight pregnancies

^c^Seven women delivered 11 babies, three women delivered twice

^d^Eight women delivered 10 babies

^e^Three women had eight pregnancies

^f^Four women had six pregnancies

^g^Four studies did not specifically report the number of attempted conceptions; in these cases, the authors considered the total number of patients who underwent successful FST

**Table 5:** NACT fertility outcomes by surgical approach

Outcomes	Cone	VRT	LRT	ART
Pregnancy rate	41.9 (13/31)	55.0 (22/40)	50.0 (2/4)	20.0 (3/15)
Birth rate	45.1 (14/31)	50.0 (20/40)^a^	50.0 (2/4)	40.0 (6/15)
Preterm rate	61.5 (8/13)	36.0 (9/25)	25.0 (1/4)	0 (0/6)

Data are expressed as % (n/N)

*NACT* neoadjuvant chemotherapy, *VRT* vaginal radical trachelectomy, *LRT* laparoscopic trachelectomy, *ART* abdominal radical trachelectomy

^a^15 women delivered 21 babies

## Discussion

Cervical cancer still represents one of the most frequently diagnosed cancers worldwide and the fourth leading cause of cancer death in women.^23^ In the two most recent decades, there has been an increase in patients in their childbearing years diagnosed with ECCs due to the widespread use of cervical cancer screening programs. In this scenario, preserving fertility remains a crucial challenge to gynecological oncologists. Tumor size is an important prognostic factor to outline the ideal candidate for FSTs and leads to a clinical approach. In fact, National Comprehensive Cancer Network (NCCN) guidelines^24^ recommend fertility-sparing surgery as an option for reproductive-aged women with stage IB1 disease, and emphasize that this approach is most validated in lesions < 2 cm in size. To date, this group of patients can benefit from several surgical techniques to maintain their reproductive potential. These methods include a simple conization to RT with and without lymphadenectomy,^25,26^ according to general indications for ECC. RT has evolved significantly over the years and several different approaches are available: vaginally, abdominally, or laparoscopically/robotically.^25^ When several procedures seem to offer the same oncologic outcomes, it is crucial to find an acceptable compromise between the best choice of cure and fertility results. VRT or conization/simple trachelectomy have shown encouraging results regarding safety and pregnancy rate.^2,25^ Much more debatable is which strategy to adopt in the case of ECC ≥ 2 cm.^26^ In these patients, VRT is contraindicated due to the high risk of recurrence^27^ and two main strategies have been proposed: abdominal surgical FST or NACT FST.^28^ In a previous review, our group collected the literature evidence regarding managing this type of patient, focusing on oncological outcomes.^5^ The results of this work ended in extremely heterogeneous data that reflect current clinical practice. Nevertheless, approaches limited to minimally invasive or vaginal techniques seem to show the highest recurrence rate (RR)^5^ and ART seems to be a safer option, according to recent evidence from the LACC trial.^28^ On the other hand, some literature reported that despite this oncological safety, ART proved to result in worse pregnancy results.^27^ In the reported series, surgical FST showed a pregnancy rate of between 12.5 and 50%, and only Cao et al. ^8^ published data on fertility outcomes in patients treated with ART or VRT. The authors confirmed that RR was higher in the VRT group (*p* = 0.040), and in four of seven recurrences, the recurrent sites after VRT were found to be located in the parametrical tissue. Hence, ART could be a safe option for patients with ECC > 2 cm, but this result does not mean it is the best choice to preserve fertility potential. Obstetric results in ART FST were not encouraging, with a pregnancy rate of 20%.^29^ Our results agree, showing pregnancy and birth rates of 22 and 11%, respectively.

Several factors can affect fertility after ART. First, a higher risk of adhesion^29^ or a higher frequency of septic morbidities linked to an abdominal approach. The lower fertility rate after a laparotomic RT could also be related to greater disruption of pelvic nerve innervations and abnormalities of the fallopian tubes. In addition, ART is usually performed with ligation of the uterine arteries that theoretically impact on fertility.^30^ Nevertheless, a subanalysis conducted by Bentivegna et al. of 735 cases showed that the infundibulopelvic and ovarian vessels could supply the vascular network of the uterine corpus, allowing a pregnancy to be achieved.^31^ An innovative approach that can extend the possibility of an FST in women with ECC > 2 cm was NACT. In this work, we reported the fertility results of 90 patients treated with NACT followed by surgical procedures (simple conization, ART, or VRT). The pregnancy and birth rates were higher compared with those observed after an upfront RT, i.e. 44 and 45% versus 22% and 11 (*p* < 0.001). Furthermore, it should be pointed out that in the NACT group, some authors reported high pregnancy and birth rates in patients with the use of conization or VRT after NACT.^9,17^

This is easily understood if we focus on the surgical implications on fertility. The use of NACT is conceived to minimize surgical aggressiveness. Combining NACT with ART means adding the surgical impact of pelvic anatomy to chemotherapeutic damage to the ovaries. However, patients treated with simple conization or VRT did not present optimal fertility outcomes. This finding can be partly explained by considering that the leading cause of obstetrical failure is related to cervical stenosis,^32^ lack of cervical mucus, and the length of the cervix or isthmus. On the other hand, the gonadotoxicity exerted by chemotherapy should be mentioned. Drugs such as platinum and paclitaxel are considered at intermediate risk of gonadotoxicity.^33^ There are strategies to minimize gonadotoxic damage using gonadotropin-releasing hormone (GnRH) agonists that decrease the risk of premature ovarian failure (POF).^33^ Unfortunately, none of the studies in the literature provided information regarding the use of these treatment regimens, which should be considered the optimum to ensure the best chance of preserving patients’ fertility. While less radical surgery is a definite trend for ECC < 2 cm,^34^ supported by a poor risk of parametria spread^35^ in patients with tumors > 2 cm could be a risky strategy. Conceptually, in selected patients treated with NACT, chemotherapy responders with no residual diseases, less radical surgery could be a reasonable approach to improve obstetric outcomes once negative lymph node status has been assessed. This leads to another controversial point related to NACT and fertility preservation—the time of lymphadenectomy. Some authors prefer to perform lymphadenectomy before administering chemotherapy,^9,12,18,19,36^ excluding node-positive patients from NACT because of the high risk of recurrence. On the other hand, post-NACT staging could have advantages in terms of no delays in treatment initiation and the possibility to sterilize lymph node micrometastasis in patients who would otherwise be excluded from the procedure.^14–16^

Similarly, in patients with ECC ≥ 2 cm, the modalities of lymphadenectomy are also controversial. Despite the high risk of lymph node metastasis, using the sentinel lymph node (SLN) could minimize the risks of lymphadenectomy-related morbidity^37,38^ and provide information on the presence or absence of micrometastases by ultrastaging.^39^ The upstream intent would be to identify patients with positive lymph nodes to exclude them from the FST pathway, regardless of the ART or NACT approach. Therefore, we believe systematic or SLN-limited lymphadenectomy should precede FST and be part of the standard diagnostic pathway of patients with ECC ≥ 2 cm.

Another consideration to be made relates to the pregnancy rate. No studies, regardless of approach, have reported on whether or not patients were directed to specialized in vitro fertilization (IVF) centers. Cancer patients, all the more so if they have undergone NACT cycles, need to be assisted in their procreation journey. On the other hand, patients should be framed from a fertility point of view before being referred to FST. None of the reported studies performed an anti-mullerian hormone (AMH) assay prior to FST. Currently, the main guidelines^24^ give 40 years of age as the limit to FST, which may not reflect the patient’s reproductive capacity at all. This biased view of the problem is perhaps related to the specifics of individual teams, which, dealing primarily with oncologic pathology, may need to be more trained in obstetrics and medically assisted procreation issues. Therefore, FST treatments should be multidisciplinary.

Finally, it is worth considering that in this review, the overall birth rate of 14.4 % is related to a preterm rate of 37%. In particular, premature delivery is often caused by PROM,^9,12,15^ likely caused by clinical or subclinical chorioamnionitis. Hence, although the fertility outcome is promising, premature birth or first-trimester fetal loss remains a main problem.^40^ The main explanation is likely related to a shortened cervix length and potential exposure of the amniotic membrane to the bacteria of the vagina, which can lead to an increase in infections. The literature reported several strategies to decrease this risk, such as prophylactic cerclage^10,14^ and the Saling procedure,^41^ a total occlusion of the uteri cervix using vaginal mucosa. Vice versa, considering cerclage might result in bladder irritation, pelvic infection, and stenosis,^42,43^ some groups abandoned performing a prophylactic cerclage and preferred to monitor the length of the cervix during pregnancy using TVU^44,45^ and placed a cerclage when necessary.^10^ However, a routine cerclage during ART may justify the lower percentage of preterm births in the surgical group of patients, even if the low number of births makes it obligatory to look at these data with skepticism.

Undeniable is that when a pregnancy occurs in women who underwent an FST, this pregnancy is at high risk. A standardized follow-up modality should be applied to improve obstetrical outcomes in pregnant women after FST.

In addition, it is interesting to note that considering only series with available data, only 35% of women who completed FST tried to conceive during follow-up. In their work, Carter et al. showed that many women who have undergone an RT experience distress that persists for up 6 months in terms of sexual disorders. In fact, pregnancy concerns appear to increase after FST, leading to lower fertility outcomes. However, studies investigating factors that affect a women’s choice to conceive are lacking, underestimating a crucial aspect of the physical and emotional impact on patients undergoing FST. Future studies in this area are needed to offer these women more complete and personalized counseling before treatments.

Fertility outcomes should only be considered in light of the comparable oncological safety of the different techniques. This could be the truth for ART and NACT, as previously published by our group.^5^

The strengths of this study lie in its systematic nature and rigor of the research, collecting the largest number of FSTs in patients with ECC >2 and adding the most possible information on obstetrical outcomes. However, the main weakness of the study is that most of the analyzed series focused on oncological outcomes, and only some of them detailed the total number of patients wishing to become pregnant, as well as every detail about each pregnancy. It almost seems that fertility outcomes have always been regarded as secondary to oncological outcomes. This is understandable in the hierarchy of these concepts, but makes it difficult to obtain standardized information on the mode of conception, obstetric care, and mode of delivery. All three confounders have implications in fertility outcomes.

## Conclusion

Fertility-sparing treatment in patients with ECC ≥ 2 cm remains a challenge, especially considering the significant heterogeneity in clinical management. This becomes even more challenging when the point of evaluation in best treatments should include oncological and obstetrical outcomes together. Nevertheless, NACT followed by minimally invasive surgery seems to be a reasonable compromise, from an obstetrical point of view. Still, standardization of treatments remains a distant goal due to the many factors involved in evaluating these patients. Moreover, guidelines on the management of pregnancy after FST are lacking and future studies are needed to investigate the best strategy to reduce the high risk of preterm delivery and PROMs.

## Supplementary Information

Below is the link to the electronic supplementary material.

Supplementary file1 (DOCX 18 kb)
