# Supplementary material for: Fertility-Sparing Treatment for Early-Stage Cervical Cancer ≥ 2 cm: Can One Still Effectively Become a Mother? A Systematic Review of Fertility Outcomes
Source: Ann Surg Oncol. 2023 Jun 1;30(9):5587–96. doi: 10.1245/s10434-023-13542-z (PMC10409841; doi:10.1245/s10434-023-13542-z)
Supplement: Supplementary file 1 — Supplementary file1 (DOCX 18 kb) [file 10434_2023_13542_MOESM1_ESM.docx]

1. **Supplementaries Newcastle–Ottawa scale**

| Single Arm Studies | | | | | | |
| --- | --- | --- | --- | --- | --- | --- |
| **Name** | **Country** | **Study design** | **Selection** | **Comparability** | **Outcome** | **Tot** |
| **Cao D.**  **2013** | China | Prospective  Case-control  Multicentric  Study | 3 | 2 | 1 | 6 |
| **De Vincenzo 2021** | Italy | Retrospective  Observational  Monocentric  Study | 3 | 0 | 3 | 6 |
| **Deng X.**  **2017** | China | Retrospective  Observational  Monocentric  Study | 3 | 0 | 3 | 6 |
| **Guo J.**  **2019** | China | Retrospective  Observational  Monocentric  Study | 3 | 0 | 3 | 6 |
| **Kim J.**  **2010** | Korea | Retrospective  Observational  Monocentric  Study | 3 | 0 | 3 | 6 |
| **Lanowska M. 2014** | Germany | Retrospective  Observational  Monocentric  Study | 3 | 0 | 2 | 5 |
| **Li J.**  **2011** | China | Retrospective  Observational  Monocentric  Study | 3 | 0 | 3 | 6 |
| **Lintner B.**  **2013** | Hungary  UK  USA | Retrospective  Observational  Multicentric  study | 3 | 0 | 3 | 6 |
| **Lu Q.**  **2014** | China | Retrospective  Observational  Multicentric  study | 3 | 0 | 2 | 5 |
| **Marchiole P. 2018** | France | Retrospective  Observational Monocentric study | 3 | 0 | 2 | 5 |
| **Rendón G. 2021** | Colombia | Retrospective  Observational  Monocentric  Study | 3 | 0 | 3 | 6 |
| **Robova H. 2014** | Czech Republic | Retrospective  Observational  Monocentric  study | 3 | 0 | 2 | 5 |
| **Salihi R.**  **2015** | Belgium | Retrospective  Observational  Monocentric  Study | 3 | 0 | 3 | 6 |
| **Tesfai F.**  **2020** | Netherlands | Retrospective  Observational  Monocentric  study | 3 | 0 | 3 | 6 |
| **Wethington S. 2013** | USA | Retrospective  Observational  Monocentric  Study | 3 | 0 | 2 | 5 |
| **Zusterzeel P. 2020** | Netherlands | Retrospective  Observational  Monocentric  Study | 3 | 0 | 3 | 6 |
